# Taxonomic Distribution and Molecular Evolution of Mytilectins

**DOI:** 10.3390/md21120614

**Published:** 2023-11-27

**Authors:** Marco Gerdol, Daniela Eugenia Nerelli, Nicola Martelossi, Yukiko Ogawa, Yuki Fujii, Alberto Pallavicini, Yasuhiro Ozeki

**Affiliations:** 1 Department of Life Sciences, University of Trieste, Via Licio Giorgieri 5, 34127 Trieste, Italy; 2 Graduate School of Pharmaceutical Sciences, Nagasaki International University, 2825-7 Huis Ten Bosch, Sasebo 859-3298, Japan; 3 Graduate School of NanoBio Sciences, Yokohama City University, 22-2 Seto, Kanazawa-ku, Yokohama 236-0027, Japan

**Keywords:** marine invertebrates, innate immunity, glycan-binding, lectins, pore-forming, β-trefoil

## Abstract

R-type lectins are a widespread group of sugar-binding proteins found in nearly all domains of life, characterized by the presence of a carbohydrate-binding domain that adopts a β-trefoil fold. Mytilectins represent a recently described subgroup of β-trefoil lectins, which have been functionally characterized in a few mussel species (Mollusca, Bivalvia) and display attractive properties, which may fuel the development of artificial lectins with different biotechnological applications. The detection of different paralogous genes in mussels, together with the description of orthologous sequences in brachiopods, supports the formal description of mytilectins as a gene family. However, to date, an investigation of the taxonomic distribution of these lectins and their molecular diversification and evolution was still lacking. Here, we provide a comprehensive overview of the evolutionary history of mytilectins, revealing an ancient monophyletic evolutionary origin and a very broad but highly discontinuous taxonomic distribution, ranging from heteroscleromorphan sponges to ophiuroid and crinoid echinoderms. Moreover, the overwhelming majority of mytilectins display a chimera-like architecture, which combines the β-trefoil carbohydrate recognition domain with a C-terminal pore-forming domain, suggesting that the simpler structure of most functionally characterized mytilectins derives from a secondary domain loss.

## 1. Introduction

Lectins are a large class of proteins nearly ubiquitously found in all living organisms, ranging from unicellular prokaryotes to complex multicellular eukaryotes, which play a pivotal role in mediating carbohydrate–protein interactions. These glycan-binding molecules display a remarkable molecular diversity, due to the presence of distinct Carbohydrate Recognition Domains (CRDs) that exhibit specificity for carbohydrate moieties. The extraordinary breadth of recognized ligands allows lectins to mediate fundamental biological processes, from cell adhesion [1], to cell–cell recognition [2], apoptotic cell clearance [3], embryogenesis [4], food particle recognition [5], and the discrimination between “self” and “nonself”. In this context, lectins have a paramount importance for pathogen recognition, which occurs thanks to the specific detection of molecular patterns associated with invading pathogenic agents, such as bacteria and viruses, the so-called Microbe Associated Molecular Patterns (MAMPs) [6]. This carbohydrate-dependent immune recognition mechanism elucidates how lectins participate in innate immune responses, triggering the activation of downstream immune effectors upon the recognition of exogenous entities. In organisms devoid of an immunoglobulin- and T-cell receptor-based adaptive immune system, lectins gain a primary role as a first barrier to prevent microbial invasion. Hence, it is not surprising that a significant number of lectin families underwent massive expansion during evolution in different lineages, fueled by tandem gene duplication events and subsequent fast molecular diversification (and functional specialization) via positive selection [7,8,9,10,11].

R-type lectins (RTLs), which take their name from the plant toxin ricin, are one of the many different structural superfamilies of lectins that have been described to date. These lectins are characterized by a CRD that displays a unique β-trefoil structural organization, consisting of three homologous subdomains, which most likely derive from the duplication of an ancestral smaller glycan-binding peptide [12]. Although this CRD is often found associated with other domains in large proteins that carry out functions linked with carbohydrate biosynthesis and metabolism [13], several RTLs displaying a simple architecture, which only include a single CRD, have been previously described in a few phyla of invertebrate animals [14,15,16,17]. Among these, a group of sequences that display a highly divergent primary sequence from all other previously described RTLs have attracted significant attention due to their glycan-binding specificity, which could make them interesting targets for biotechnological applications. These molecules, which are collectively known as mytilectins from the name of MytiLec-1 [18], have been so far functionally characterized only in a small group of bivalve mollusks, all belonging to the family Mytilidae [19,20,21,22,23]. Due to their marked binding specificity for globotriose (Gb3), a glycan expressed at high levels by Burkitt lymphoma Raji cells, as well as due to the cytotoxic effect exerted upon binding on these and other types of cancer cells [24,25,26] and the ability to modulate macrophage activity in mice [27], mytilectins may find practical applications in the context of cancer diagnosis and treatment. For this reason, mytilectins have been the subject of mutagenesis studies aimed at better understanding their structure–function relationships to allow the design of molecules with improved glycan-binding properties [28,29], and have been also used as a template for the in silico design of the synthetic lectin Mitsuba [30].

While all functionally characterized mytilectins display a high primary sequence homology with each other, being characterized by the presence of a single CRD, further studies have revealed the existence of additional members of the same family in mussels. These sequences display a higher molecular weight, due to the presence of a C-terminal domain that shares a striking structural resemblance with aerolysin, a cytolytic toxin from *Aeromonas hydrophila* [25]. This observation would suggest that these mytilectins, called “chimera-type” to differentiate them from the aforementioned “proto-type” mytilectins, may be involved in the formation of pores in target membranes through the formation of oligomeric beta-barrels, as happens in other toxins that have acquired similar structural features in a convergent manner [31,32,33].

More recently, sequences sharing high primary sequence homology with mytilectins have been described in *Lingula anatina*, a marine invertebrate belonging to the phylum Brachiopoda, distantly related with bivalve mollusks, implying a shared ancestry for these molecules and strongly suggesting a taxonomic spread much broader than originally thought [34]. Although mytilectins have been previously referred to as members of a novel lectin family [35,36], the lack of any specific investigations concerning their evolutionary origin, taxonomic spread, and relationships has prevented, to date, a formal description of the “mytilectin family”. Here, with a comprehensive screening of available genomic and transcriptomic resources, we provide a clear overview concerning these aspects, supporting a monophyletic origin for mytilectins deeply rooted in the metazoan lineage, and reveal that these lectins are present in an unexpectedly large number of animal phyla.

## 2. Results and Discussion

### 2.1. Taxonomic Distribution of Mytilecins

**Figure 1:**
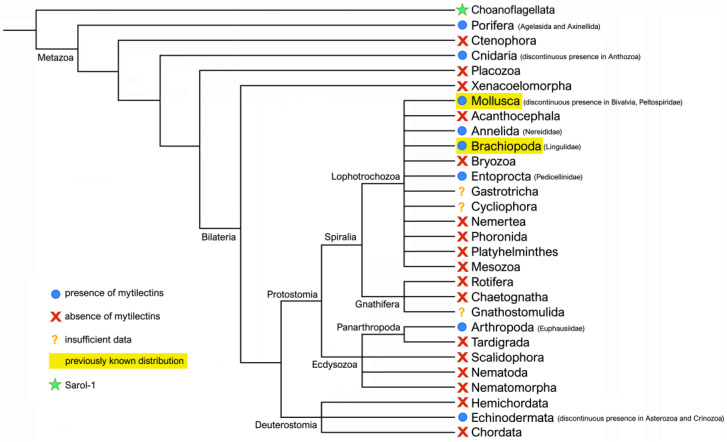
Schematic view of the distribution of mytilectins across the metazoan tree of life. Presence or absence is displayed at the phylum level, with further indication of their distribution at lower taxonomic ranks, whenever relevant. In this representation, presence indicates that some (but not all) the members of a given phylum had mytilectin genes, whereas absence indicates that no mytilecin sequences could be detected in any of the genomes or transcriptomes available for a given phylum. In a few cases, evidenced with a question mark, available genomic data was insufficient to draw definitive conclusions concerning the presence or absence of mytilectins. The phylogenetic placement of the phylum Choanoflagellata at the base of the tree is marked due to the detection of SaroL-1, a lectin sharing striking structural similarity with mytilectins, as discussed in Section 2.6.

The large-scale screening of available metazoan–omic resources allowed the authors to significantly expand the taxonomic range of distribution of mytilectins compared with previous reports, as schematically displayed in Figure 1. The presence of members of this lectin family in largely divergent animal phyla, ranging from Porifera to Echinodermata, suggest an ancient evolutionary origin predating the acquisition of bilateral symmetry. At the same time, mytilectins display a highly discontinuous distribution, characterized by their absence in several major phyla. This situation could be consistent with two alternative and not mutually exclusive scenarios, namely (i) the very ancient origin of this lectin family, preceding the acquisition of bilateral symmetry, followed by multiple independent gene loss events occurring in different taxa; or (ii) a more recent origin traceable to a single phylum, followed by several horizontal gene transfer events, which greatly expanded the narrow original distribution of this lectin family. The plausibility of these two hypotheses, in light of the recent report of the presence of SaroL-1, a lectin sharing striking structural similarity with mytilectins, in the choanaoflagellate *S. rosetta* (Figure 1), will be discussed after the comprehensive overview of the results of the comparative genomics analyses conducted in this study outlined in the next sections.

#### 2.1.1. Phylum Porifera

The most early branching metazoan phylum where mytilectins were detected was Porifera. However, mytilectin genes were only present in a single one out of the 10 sponge genomes available to date (as of November 2023), i.e., *Agelas oroides*, belonging to the order Agelasida. This finding, together with the absence of orthologous sequences in other genomes of the five species placed in the subclass Heteroscleromorpha (class Demospongiae), allowed the authors to infer the lack of mytilectins in the orders Haplosclerida, Spongillida, and Suberitida. However, further analysis of transcriptome data extended the distribution of poriferan mytilectins to the order Axinellida (in detail, mytilectin transcripts were found in *Eurypon* sp. 2 AS-2020 and *Hymeraphia stellifera* [37]). No evidence supporting the presence of mytilectins could be collected in the three other extant classes of Porifera, i.e., Calcarea, Hexactinellida, and Homoscleromorpha.

#### 2.1.2. Phylum Cnidaria

In the phylum Cnidaria, the presence of mytilectins was restricted to the class Anthozoa, where this gene family was represented across a broad range of orders belonging to two out of three subclasses (Hexacorallia and Octocorallia; no genomic data is available for Ceriantharia), with significant gaps across phylogeny, suggesting a complex evolutionary history characterized by multiple independent losses. Namely, mytilectin genes were detected in 7 out of 85 available genomes of Hexacorallia (8% of the total), i.e., *Orbicella faveolata*, *Montipora capitata* [38], *Montipora* sp. Colony 1 RG-2022, *Stylophora pistillata* [39], *Palythoa heliodiscus* [40], *Palythoa grandis* [40], and *Ricordea florida*. A few additional species could be added to this list (*Acropora tenuis* [41], *Montipora digitata* [42], *Alveopora japonica* [43], *Parachrysogorgia stellata* [44], and *Fimbriaphyllia ancora* [45]) thanks to transcriptomic evidence. Most of these species belong to different families within the order Scleractinia (i.e., Acroporidae, Chrysogorgiidae, Euphylliidae, Faviidae, Merulinidae, and Pocilloporidae), but matches were also found in Corallimorpharia and Zoantharia.

The prevalence of mytilectins in available octocorallian genomes (2 out of 11) was similar to that outlined above in Hexacorallia, with positive hits in *Paramuricea clavata* [46] and *Eunicella verucosa* [47], both classified within the order Malacalcyonacea. Transcriptome data, besides further matches in *Eleutherobia rubra* [48], *Clavularia* sp. cla_tr77125 [49], and *Scleronephthya gracillima*, expanded the range of distribution of cnidarian mytilectins to Scleralcyonacea (i.e., *Heliopora coerulea* [50]).

No evidence supporting of the presence of mytilectins could be collected in the other cnidarian classes (i.e., Cubozoa, Hydrozoa, Myxozoa, Scyphozoa, and Staurozoa), neither at the genomic nor at the transcriptomic level.

#### 2.1.3. Phylum Mollusca, Class Bivalvia

The class Bivalvia was the taxonomic group with the highest number of mytilectin sequences identified, in part due to its high species richness and abundance of genomic and transcriptomic resources, in part due to the fact that several species displayed multiple paralogous gene copies.

Fully sequenced genomes are still lacking for the subclass Protobranchia, an early offshoot of the Bivalve lineage. However, transcriptomic evidence supports the presence of mytilectins in *Solemya velum* [51] and *Ennucula tenuis* [52], which belong to two different protobranch orders, i.e., Nuculida and Solemyida. This finding indicates the likely presence of a mytilectin gene in the latest common ancestor of all bivalves. As far as the second bivalve subclass (i.e., Autobranchia), is concerned, mytilecin genes were identified both in the infraclass Pteriomorphia and in the infraclass Heteroconchia. Nevertheless, the distribution of mytilectins was sparse with significant gaps, mirroring the general situation outlined at higher taxonomic ranks in Figure 1.

According to the current WoRMS classification, Pteriomorphia includes five orders: Arcida, Limida, Mytilida, Ostreida, and Pectinida. Mytilectins were clearly missing in Ostreida, as no significant homology could be detected in any of the 14 fully sequenced genomes available to date. Although no genome data was available for Limida, a similar conclusion could be drawn for this order based on the analysis of transcriptome data [53]. On the other hand, mytilectins were present in one out of the two genomes of Arcida, i.e., *Tegillarca granosa* [54]. Mytilectins were present in the genomes of 6 out of 13 species belonging to the order Mytilida. Five of these (*Mytilus californianus*, *Mytilus chilensis*, *Mytilus coruscus*, *Mytilus edulis,* and *Mytilus galloprovincialis*) [55,56,57,58,59] are congeneric, confirming the previous reports of MytiLec-1, CGL, and MTL in *Mytilus* and *Crenomytilus* spp. [20,26,60]. The sixth species was *Perna viridis* [61], which also belongs to the subfamily Mytilinae. Mytilectin-encoding transcripts were also detected in the congeneric species *Perna perna* [62] and *Perna canaliculus*. The transcriptome of the ribbed mussel *Geukensia demissa* [63] allowed the authors to expand the range of distribution of mytilectins to a second mytilid family, i.e., Brachidontinae. Mytilectins were widespread in Pectinida, as evidenced by their presence in all the six species with a fully sequenced genome available (*Pecten maximus*, *Mizuhopecten yessoensis*, *Argopecten irradians*, *Argopecten purpuratus,* and *Mimachlamys varia*) [64,65,66,67,68] and by their detection in the transcriptomes of *Adamussium colbecki* and *Nodipecten subnodosus* [69,70].

In the infraclass Heteroconchia, mytilectins were clearly absent in the genomes of all Palaeoheterodonta, which include the large majority of freshwater mussels and clams, and likely absent also in Archiheterodonta. Despite being absent in the superorder Anomalodesmata, mytilectins were found in 5 out of 24 available fully sequenced genomes available for the superorder Imparidentia, part of Euheterodonta, the largest group of heteroconch bivalves. Three of these were members of the order Myida (*Congeria kusceri*, *Dreissena polymorpha,* and *Mya arenaria*) [71,72] and the other two (*Mercenaria mercenaria* and *Saximodus purpurata*) [73] belonged to the order Venerida. Nevertheless, several other species classified as venerid clams lacked mytilectin genes. The abundance of available genomes for the order Cardiida allowed ruling out the presence of mytilectins in this taxa. Although -omic resources are still limited for other minor Imparidentia orders, neither genomic, nor transcriptomic data supported the presence of mytilectins, with the lone exception of a partial transcript sequence detected in *Hiatella arctica* (order Adapedonta) [53].

#### 2.1.4. Other Mollusca

Despite their frequent occurrence in Bivalvia, mytilectins were absent in the overwhelming majority of other mollusks. The lone exception was represented by the three species of the family Peltospiridae (class Gastropoda) with a sequenced genome available, i.e., *Chrysomallon squamiferum* [74], *Dracogyra subfusca,* and *Gigantopelta aegis* [75], which are all deep-sea hydrothermal vent snails. The lack of genomic resources for sister taxa did not allow the authors to ascertain whether mytilectins were restricted to Peltospiridae or more broadly distributed in all the members of the order Neomphalida. Although over 50 other gastropod genomes have been sequenced and assembled to date, none of these presented mytilectin-encoding genes, indicating a highly reduced representation of this gene family in the most species-rich molluscan class.

Albeit significant taxonomic gaps are still present for the minor molluscan classes (e.g., Monoplacophora, Scaphopoda, and Caudofoveata), no evidence supporting the existence of mytilectins could be found in Solenogastres, Cephalopoda, and Polyplachophora.

#### 2.1.5. Other Lophotrochozoa

The presence of mytilectins was previously reported in *Lingula anatina*, a member of the phylum Brachiopoda [34]. Although this species remains to date the only brachiopod with a sequenced genome, current evidence suggests that mytilectin distribution in this phylum might be very narrow, due to the lack of orthologous sequences in the transcriptomes of several other brachiopod species.

The only other lophotrochozoan phylum with compelling evidence supporting the presence of mytilectins was Annelida. In detail, matches were identified, either at a genomic or a transcriptomic level, in the family Nereididae, consisting of polychaete worms (subclass Errantia, order Phyllodocida). Namely, mytilectin sequences were found in *Alitta virens* [76], *Perinereis aibuhitensis* [77], and *Platynereis dumerilii* [78]. The absence of mytilectins in several genomes belonging to the same order, but different families, points to a distribution restricted to nereidid polychaete worms.

The only other instance of detection of partial sequences with a clear homology with mytilectins was *Pedicellina cernua* (Entoprocta, Pedicellinidae) [79]. However, the lack of –omic resources for this neglected minor lophotrochozoan phylum impeded further confirmation of this finding at the genome level and the exploration in other species belonging to closely related taxa.

#### 2.1.6. Ecdysozoa

Mytilectins were largely absent in Ecdysozoa, which include the most species-rich group of animals, i.e., arthropods. In fact, only a single out of the nearly 2700 arthropod genomes available to date (most of which are from Insecta) carried mytilectin genes. This surprising finding was made in the Antarctic krill *Euphausia superba*, whose recently sequenced genome is one of the largest ever reported in the animal kingdom [80]. Further transcriptomic investigations confirmed this finding and allowed the auhtors to detect mytilectins in the transcriptome of a second species belonging to the family Euphausiidae, i.e., *Meganyctiphanes norvegica* [81]. The absence of sequences bearing detectable homology in the genomes of about 90 other crustacean species clearly marked the restricted presence of mytilectins in krill as a unique case in Ecdysozoa.

#### 2.1.7. Phylum Echinodermata

Within Deuterostomata, Echinodermata was the only phylum where mytilectin sequences could be unambiguously identified and confirmed both at a genomic and at a transcriptomic level. Nevertheless, like other previously described cases, mytilectins were detected just in a restricted number of taxa.

In detail, mytilectin genes were found only in two out of the three extant echinoderm subphylums, i.e., Asterozoa and Crinozoa, thereby pointing out their absence in both sea urchins and sea cucumbers. The presence of mytilectins could be only confirmed in a subgroup of asterozoans, i.e., those belonging to the class Ophiuroidea. In detail, complete or partial sequences were recovered in *Amphiura filiformis* [82], *Ophiothrix exigua* [83], *Ophiothrix spiculata* [84], and *Ophioderma brevispina* [85]. Albeit these four species belong to the subclass Myophiuroida, the lack of genomic data for the other ophiuroid species prevented a more precise assessment of taxonomic delimitation in this case. Mytilectin genes were also detected in a single crinoid species, i.e., *Nesometra sesokonis* [86], and further investigations failed to identify orthologous sequences in the transcriptomes of several other crinoids, suggesting a narrow taxonomic distribution within this subphylum.

#### 2.1.8. Other Deuterostomes

No significant matches supporting the presence of mytilectins were found in the genomes of deuterostome phyla other than Echinodermata. These comprised all representatives of the phyla Hemichordata and Chordata, including cephalochordates and tunicates. This was strongly supported by transcriptomic evidence, even though a few assembled transcripts linked to *Sardinops melanostictus* (Actinopterygii) matching mytilectins are present in the TSA repository. However, upon further inspection of their phylogenetic placement, these sequences were found to bear close similarity with bivalve mytilectins. Taking into account that bivalve larvae are usual components of the diet of sardines [87], we could safely classify this finding as a false positive linked to the ingestion of an unidentified bivalve species by the fish.

### 2.2. Structural Features of Mytilectins

**Figure 2:**
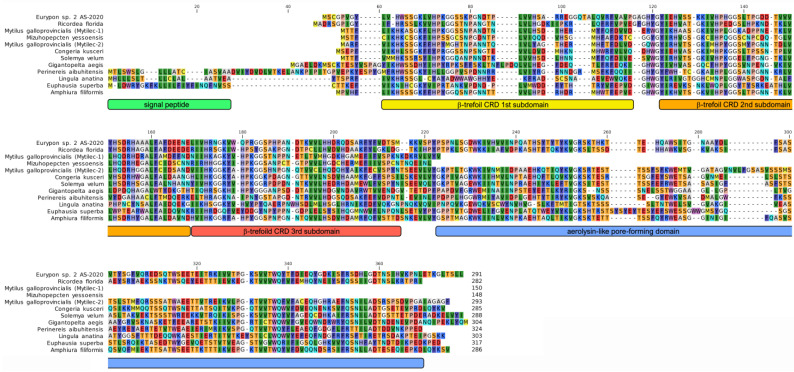
Multiple sequence alignment of representative mytilectins from different phyla. The most significant structural features discussed in the text (i.e., the presence/absence of a signal peptide, the delimitation of the CRD, and pore-forming domain) are highlighted. Note that the *M. galloprovincialis* MytiLec-1, and the *M. yessoensis* sequences reported in the MSA are proto-type mytilectins.

We had previously described the presence of two distinct types of mytilectins [88], defined as “proto-type” and “chimera-type”, respectively, characterized by markedly different length and domain architecture. Proto-type mytilectins, which include all functionally characterized mytilectins to date (i.e., CGL, MTL and MytiLec-1), display a single β-trefoil CRD with no accessory domains, being approximately 150 aa long. On the other hand, other mytilectins identified in *M. galloprovincialis* and *L. anatina* were significantly longer due to the presence of a C-terminal extension encoding an additional 140 aa-long domain, whose three-dimensional structure was predicted to resemble that of *Aeromomas hydrophyla* aerolysin and other pore-forming toxins [34,89]. The presence of these two distinct domain architectures leaves an open question concerning the ancestral structural configuration of mytilectins, due to the existence of two alternative evolutionary scenarios: indeed, the CRD/pore-forming domain combination observed in chimera-type mytilectins could be the result of a gene fusion event involving an ancestral proto-type mytilectin gene, or, alternatively, proto-type mytilectins could be the result of the secondary loss of the pore-forming domain present in the ancestral chimera-type gene. To provide an answer to this question, we collected over one hundred mytilectin sequences from multiple phyla, significantly expanding the repertoire of both mytilectin types. The multiple sequence alignment displayed in Figure 2 reports a representative group of the mytilectins recovered in this study.

The data we collected strongly support the second out of the two aforementioned evolutionary scenarios, due to the presence of chimera-type mytilecins in all early branching metazoan phyla, such as Porifera and Cnidaria. Moreover, chimera-type mytilectins were far more widespread than proto-type mytilectins, as the latter were only detected in *Mytilus* spp., in the brachiopod *L. anatina* and in all pectinid bivalves. Nevertheless, while both domain architectures were simultaneously present in mussels and brachiopods, scallops were the only species to uniquely display proto-type sequences. Moreover, within bivalves, proto-type mytilectins were absent in Arcida and Heteroconchia, further supporting a secondary domain loss in the proto-type mytilectins of mussels and scallops.

Another interesting structural feature which has been likely acquired in a convergent manner by distinct phyla concerns the N-terminal region of mytilectins. As previously reported [88], mussel mytilectins lack a detectable signal peptide for secretion, thereby being targeted to the extracellular environment though a non-canonical secretion pathway. This peculiarity is shared by the overwhelming majority of the mytilectins detected in this study and clearly represents the ancestral status of the mytilectin precursor gene, as suggested by the lack of signal peptides in the sequences of sponges and anthozoans (Figure 2). Nevertheless, three notable exceptions were identified: brachiopods (as previously reported in [34]), nereidid polychaetes, and krill. Indeed, these three taxa displayed well-supported signal peptides, which would support the secretion of these mytilectins following the canonical route mediated by signal recognition particles.

### 2.3. Gene Architecture Strongly Supports a Monophyletic Origin for all Mytilectins

As briefly discussed above, the highly discontinuous taxonomic distribution of mytilectins summarized in Figure 1 could be consistent with two alternative scenarios, the likelihood of which will be here evaluated using phylogenetic inference and gene architecture information. The conservation of exon/intron boundaries and splicing sites among genes from distantly related phyla is generally considered as strong evidence of orthology [90,91,92], to the point that this factor is often incorporated in gene prediction algorithms [93]. On the other hand, convergent independent intron gain in the same position, with the very same phase, is considered unlikely [94] and the presence of a reduced number of introns, or their entire lack, has been previously reported as a typical feature of eukaryotic genes recently acquired by horizontal gene transfer [95]. Hence, the detection of shared intron positions by mytilectin genes of distantly related phyla would provide strong evidence supporting an ancient monophyletic origin for mytilectins, which would be consequently inferred to be already present in the latest common ancestor of all metazoans. We will here report the intron/exon architecture of mytilectins with regard to the coding sequence only, even though some mytilectin genes most certainly include an exon in the 5’end, before the ATG start codon [88]. This is necessary due to the focus placed by standard genome annotation pipelines on coding sequences, which may therefore often entirely miss or mis-annotate 5’ and 3’ UTR regions. At the same time, we will here mostly focus on the gene architecture of proto-type mytilectins, as the genes encoding proto-type mytilectins are significantly shorter due to the lack of the pore-forming domain (see Section 2.2).

**Figure 3:**
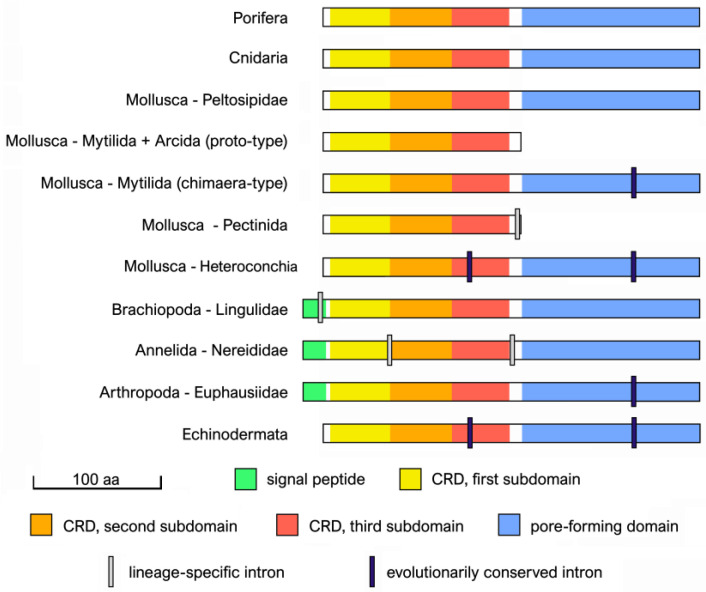
Schematic view of the architecture of mytilectin genes in the major animal taxa where this lectin family was identified. Colored bars indicate the full-length sequence of mytilectin precursor proteins, with indication of the position of introns relative to the N-terminal CRD (subdivided among the three subdomains of the β-trefoil fold) and to the C-terminal pore-forming domain. Please note that only the mytilectins of Brachiopoda, Annelida, and Arthropoda display a signal peptide, which determines a slight extension of the protein sequence at the N-terminal end. As discussed in the text, introns located in the 5’ and 3’ UTR were disregarded due to the frequent lack of accurate annotations of these regions in available genome assemblies.

The complete analysis of the available genomic data revealed that the full ORF of mytilectins was contained within a single exon in poriferan and cnidarian genes, as well as in those from Peltospiridae gastropods. Due to the basal placement of sponges and cnidarians in the animal tree of life (see Figure 1), this data would suggest that the ancestral metazoan mytilectin gene was intronless. However, mytilectin genes acquired introns in several other taxa during evolution, leading to slightly more complex splicing patterns, which may split the ORF between either two or three coding exons. Interestingly, despite the presence of lineage-specific losses and acquisitions, the placement of splicing sites was often conserved across largely divergent phyla, thereby strongly supporting a shared monophyletic evolutionary origin for animal mytilectins (Figure 3).

In detail, the ORF of chimera-type mytilectins from echinoderms (including both ophiurids and crinoids), bivalves, and krill was interrupted by the presence of an intron in the very same position, i.e., roughly breaking in two equal parts the C-terminal pore-forming domain. Echinoderms and heteroconch bivalves also shared an additional intron in the N-terminal region, within the third subdomain of the β-trefoil CRD. This exon was missing in krill, mussels, and ark shells, but also in scallops (which only have shorter proto-type mytilectin genes), which nevertheless displayed a single intron in a different position, i.e., after the end of the CRD.

Additional independent lineage-specific intron gain events could be inferred in nereidid polychaetes, which displayed two introns, placed roughly between the first and the second CRD subdomains, and in the region connecting the CRD to the pore-forming domain, respectively, and in Brachiopoda, where the ORF was interrupted close to the N-terminal end. Interestingly, the placement of this intron may explain the acquisition of a signal peptide by brachiopod mytilectins, perhaps due to exon shuffling phenomena (see Section 2.2).

### 2.4. Phylogeny of Mytilectins

**Figure 4:**
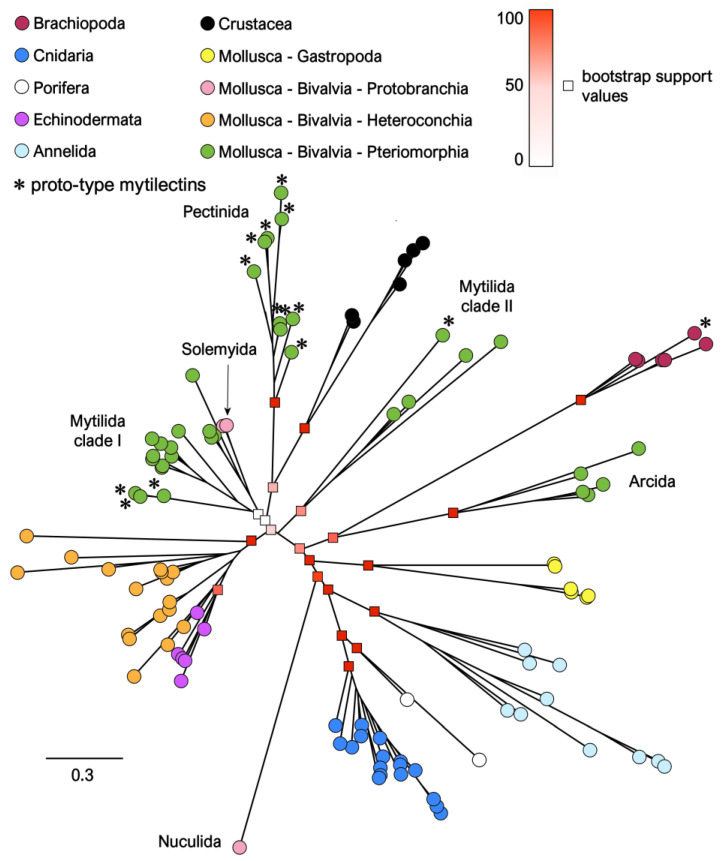
Maximum likelihood phylogeny of mytilectins, represented as an unrooted tree. Individual sequences are displayed as circles, colored based on the major taxonomic groups discussed in the main text (i.e., Brachiopoda, Cnidaria, Porifera, Echinodermata, Crustacea, and Mollusca, further subdivided among Gastropoda and three taxonomic groups of Bivalvia, i.e., Protobranchia, Heteroconchia, and Pteriomorphia). Further lower-rank classifications discussed in the main text are also indicated close to the relevant clades. For the sake of simplicity, statistical support for major basal nodes is reported with squares, whose color indicates bootstrap values. Proto-type mytilectins are marked with an asterisk.

We investigated the evolutionary relationships among the mytilectins identified in this study through maximum likelihood phylogenetic inference, revealing a complex picture which did not fully mirror the well-established taxonomic placement of the taxa where mytilectin genes were present (Figure 4).

For example, even though sponge and cnidarian sequences were placed in the same monophyletic clade with maximum statistical support (bootstrap value = 100), thereby reflecting the early branching position of these phyla in the animal tree of life, neither protostome nor deuterostome mytilectins were grouped in monophyletic clades, as would have been expected in the case mytilectin evolution closely following the evolutionary relationships among species. Nevertheless, while considering lower taxonomic ranks, a high number of sequence groups matched highly supported monophyletic clades: this was the case with Pectinida (bootstrap support = 100), Arcida (bootstrap support = 100), Crustacea (bootstrap support = 100), Brachiopoda (bootstrap support = 100), Annelida (bootstrap support = 100), and Gastropoda (bootstrap support = 100).

On the other hand, this was not the case for the sequences of Mytilida, which, as reported in Section 2.2, are highly diversified from a structural point of view. These sequences were indeed grouped in two clades, also highlighted in Figure 4: the poorly supported clade I (bootstrap support = 22), included a mixture of mytilid proto-type and chimera-type sequences, together with the two sequences from Solemyida (Protobranchia). On the contrary, the better supported clade II (bootstrap support = 61) exclusively included mytilid sequences (both proto- and chimera-type). A second large clade of sequences characterized by high statistical support (bootstrap support = 100) included a subgroup of bivalve sequences (i.e., those from Heteroconchia) and all echinoderm mytilectins. Although the bootstrap support for the internal nodes of this branch of the tree were low, hindering further investigation into the detailed relationships between the mytilectins of these two phylogenetically distant taxa, their high primary sequence similarity, strongly supported by ML inference, would point to an interesting case of convergent evolution.

The scattered position of proto-type mytilectins in the phylogenetic tree (marked by asterisks in Figure 4) further supports the previously hypothesized independent origin for these modified mytilectins in Mytilida, Pectinida, and Brachiopoda, as outlined in Section 2.2. Similarly, the mytilectins that display a signal peptide (i.e., those from Branchiopoda, Crustacea, and Annelida) would create a polyphyletic group, thereby strongly supporting the independent acquisition of a signal for canonical secretion in these three taxa.

Overall, the molecular phylogeny of mytilectins opens several interesting evolutionary questions, which can only be partially solved at the moment due to the current lack of information concerning the functional specialization (if any) of proto-type and chimera-type mytilectins. The significant discrepancies between gene and species phylogeny in this case may have several different explanations. Undoubtedly, mytilectins are relatively short proteins (i.e., the total size of the multiple sequence alignment analyzed with phylogenetic inference was 268 amino acids), which often display limited primary sequence homology (with p-distances in the range of 0.7–0.8 in inter–phyla pairwise comparisons), and the low number of phylogenetically informative sites may have led to the incorrect or poorly supported relative placement of some sequence groups, fundamentally altering the ordering of some nodes of the tree. Nevertheless, as highlighted above, mytilectins display several convergent features, which include the independent loss of the pore-forming domain, as well as the independent acquisition of a signal peptide in different phyla. For similar reasons, one might expect to observe the independent occurrence of other convergent sequence features, whose weight on such a short MSA may have understandably led to unexpected branching patterns. Moreover, the lack of non-metazoan sequences showing high homology with mytilectins to be used for rooting purposes, together with the low number of available mytilectins from the most basal animal group (i.e. Porifera, with just two sequences available) represented another limitation for phylogenetic inference. Finally, it needs to be considered that the unusual and highly discontinuous taxonomic distribution of mytilectins in extant species is most likely the product of massive gene loss events, which resulted in the availability of a particularly low number of sequences in a few key taxa.

### 2.5. Optimization of Mytilectin-Specific Hidden Markov Models

The β-trefoil and pore-forming domains of mytilectins share a significant structural homology with several R-type lectins and aerolysin-like toxins from multiple organisms, thereby allowing the identification of these conserved domains using Hidden Markov Models (HMM). For example, the presence of the mytilectin β-trefoil domain would allow the recognition of mytilectins as members of the Ricin B-like lectins homologous superfamily (IPR035992) in InterPro [96], as well as members of the CATH superfamily 2.80.10.50 [97]. Nevertheless, such classifications are extremely generic, as to date over 93 and 184 thousand protein sequences fit within these large superfamilies. Similarly, the pore-forming domain of chimera-type mytilectins matches the CATH superfamily G3DSA:2.170.15.10, which includes over 7000 different sequences.

Taking this into account, we investigated the possibility of generating HMM profiles that could be used to specifically identify mytilecins by separately detecting the two structural units found in chimera-type sequences. The performance of the two resulting profile HMMs, obtained as described in Section 3, and named “mytilectin N-terminal domain” and “mytilectin C-terminal domain”, respectively, was tested against the full set of mytilectins described in this manuscript and all the protein sequences deposited in UniProt.

These tests confirmed the high specificity of both HMMs. In detail, the N-terminal HMM could positively identify all mytilecins, with e-values ranging from 6.7e^−70^ to 3.6e^−31^, with the worst results usually obtained for the detection for mytilectins from polychaete worms, crustaceans, and ark shells. The C-terminal HMM was also able to identify all mytilectins, with e-values ranging from 1.7e^−54^ to 1.3e^−28^. In this case, the worst results were observed for the mytilectins of brachiopods, crustaceans, and gastropods. These good performances were accompanied by a lack of false-positive detections in UniProt: as expected, the non-target hits achieving the best scores for the two HMMs (i.e., the mucoricin RLT1_RHIO9 and the monalysin MONAL_PSEE4, respectively) belonged to the same structural superfamilies. However, their e-values (2.9 and 0.097, respectively) remained far below the detection threshold.

### 2.6. On the Occurrence of the β-Trefoil/Aerolysin-like Pore-Forming Domain Combination in Non-Metazoan Phyla: Convergent Evolution or Shared Ancestry?

Although our recursive homology search strategy (see Section 3.1) was limited to Metazoa, we noticed the presence of statistically significant similarities between a few animal mytilectins and a single sequence recently described in the choanoflagellate *S. rosetta*, belonging to a sister group of Metazoa within Opisthokonta. This sequence was Sarol-1, characterized as a pore-forming protein from this unicellular marine eukaryote in 2022 [98]. As previously noted by Notova and colleagues, the N-terminal region of Sarol-1, corresponding to the β-trefoil domain, displayed a significant homology, both at the primary sequence and at the structural level, with MytiLec-1, CGL, and MTL. Most importantly, unlike functionally characterized mytilectins, Sarol-1 also displayed a long C-terminal extension which structurally resembled aerolysin, thereby completely matching the domain architecture of chimera-type mytilectins (see Section 2.2). In light of this observation and of our novel identification of mytilectins in the phylum Porifera (see Section 2.1), one might wonder whether the evolutionary origins of mytilectins might be pushed further back deeply into the Opisthokonta lineage, at the very least to the latest common ancestor of the Metazoa and Choanoflagellata lineages.

While the remarkable primary sequence similarity (i.e., up to 30–35%) between the β-trefoil domains of Sarol-1 and some mytilectins may support this view, a much higher divergence was observed between the aerolysin domains of Sarol-1 and chimera-type mytilectins. This was fully consistent with the results obtained with the analysis of the Sarol-1 sequence with mytilecin-specific profile HMMs described in Section 2.5. Indeed, the β-trefoil domain of Sarol-1 could be recognized with a highly significant e-value (i.e., 1e^−30^), whereas the aerolysin-like domain could not be recognized at all. Hence, although Sarol-1 could be reasonably considered as a mytilectin-related sequence that most likely shares ancestry with metazoan mytilectins, its primary sequence does not fully conform with that of chimera-type mytilectins. Based on the available sequence data, it is therefore unclear whether the pore-forming domain was acquired independently in the choanoflagelate and metazoan lineages, or whether the significant divergence observed between Sarol-1 and mytilectins in this domain is due to rapid evolution that made the presence of homology unrecognizable.

**Figure 5:**
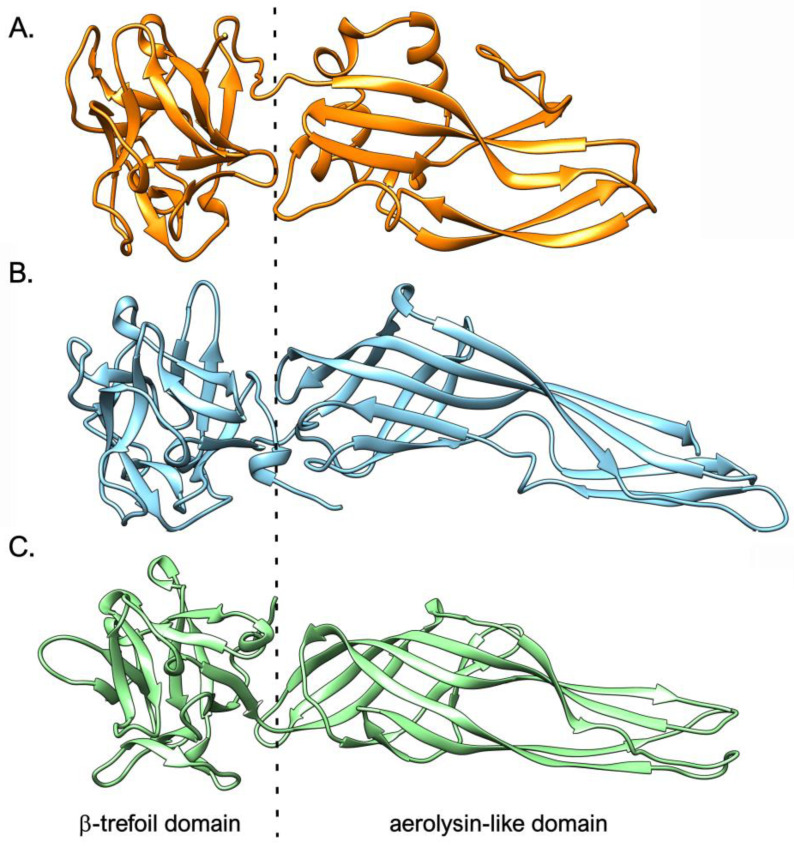
Comparison between the three-dimensional structures of *M. galloprovincialis* mytilectin-3, selected as a representative member of metazoan chimera-type mytilectins (panel (**A**)), *S. rosetta* Sarol-1 (panel (**B**), PDB ID: 7QE4), and *L. sulphureus* LSL (panel (**C**), PDB ID: 1W3F).

As a side note, it needs to be remarked that structural similarity-based detection methods, such as HHPRED [99], allowed the identification of other proteins sharing the very same domain combination of chimera-type mytilectins and Sarol-1 in other phyla, even though, in these cases, primary sequence similarity was too low to allow the detection of homology through BLAST. As highlighted by Figure 5, this was the case with LSL, a toxin produced by the mushroom *Laetiporus sulphureus* [31], and with the insecticidal toxins Tpp80Aa1 and BinAB from *Bacillus thuringiensis* and *Lysinibacillus sphaericus* [100,101]. Since the possibility of detecting such structural convergence is currently restricted to protein sequences whose three-dimensional structure has previously been experimentally determined, the number of proteins found in nature that have acquired the very same domain combination in a convergent manner is likely much higher.

## 3. Materials and Methods

### 3.1. Retrieval of Mytilectin Sequences from Public Databases

Mytilectin sequences were retrieved through a recursive homology search approach using BLASTp [102], which used the previously described sequences of *M. galloprovincialis* (MytiLec-1, -2, and -3) [18,88] and *L. anatina* [34] as initial queries. In the first round of analysis, homology searches were carried out against the NCBI nr database and all hits with e-values lower than 0.05 were extracted and manually inspected to verify the reliability of protein sequences, with particular attention to those deriving from automated gene annotation pipelines. The sequences displaying unusual features (e.g., missing N- and C-terminal ends or with significant internal gaps) were marked as suspicious and subjected to manual confirmation as briefly described below. All newly recovered sequences were clustered by pairwise similarity with CD-HIT [103] based on an arbitrary similarity threshold of 0.5. The representative sequences of each cluster were then selected as queries for a second round of sequence homology searches. The process was performed recursively until no novel hits could be recovered.

A similar strategy was used to recover mytilectin sequences from the de novo assembled transcriptomes deposited in the NCBI TSA database, using tBLASTn [102]. In this case, positive matches were translated in silico using the Expasy translate tool [104]. Incomplete sequences (i.e., those lacking the initial ATG codon or the stop codon), as well as those characterized by obvious mis-assembly or encoding identical proteins (e.g., those encoded by redundant assembled contigs) were removed.

Finally, the homology search approach was extended to all assembled genomes deposited in the NCBI Whole Genome Shotgun database, with particular attention to genomes devoid of gene annotation. Here, mytilectin genes were manually annotated, by combining the identification of High Scoring Segment Pairs (HSPs) via tBLASTn and the prediction of canonical donor and acceptor splicing sites via Genie [105]. All the amino acid sequences of the mytilectins described in this manuscript are reported in Supplementary Material S1.

### 3.2. Protein Sequence Analysis

All the protein sequences obtained as described in the previous section were subjected to signal peptide prediction with SignalP v.6.0 [106] and analyzed with InterProScan v.5 [107] to verify the identification of a complete profile HMM attributable to the Ricin B-like lectins homologous superfamily (IPR035992). The sequences that only displayed this structurally recognizable domain were classified as proto-type mytilectins, according to previously suggested nomenclature [88], whereas those displaying an additional match in the C-terminal region, consistent with the CATH superfamily 2.80.10.50 [64], were classified as chimera-type mytilectins. The reliability of all proto-type mytilectin protein sequences derived from automated gene prediction pipelines was evaluated through the comparison of these sequences with assembled RNA-sequencing data, whenever available.

The presence of structural similarities with proteins with experimentally determined three-dimensional structures deposited in the Protein DataBank (PDB) database was investigated with HHPRED [99]. The three-dimensional structure of *M. galloprovincialis* mytilectin-3 was predicted with Alphafold v2.3.0 [108], using CASP14-like settings.

### 3.3. Phylogenetic Analysis

All mytilectin protein sequences were aligned with MUSCLE [109], obtaining a multiple sequence alignment (MSA) file that was used as an input for subsequent analyses. To reduce background noise and only keep phylogenetically informative sites, poorly alignable positions were removed. In detail, the MSA was trimmed by removing the signal peptide region (whenever present) and all residues located at the N-terminal side of the CRD. Similarly, all residues located at the C-terminal side of the pore-forming domain were also removed. Moreover, all alignment positions characterized by missing data (i.e., gaps) in >50% sequences were deleted. The resulting clean MSA, including 274 positions, was analyzed with ModelFinder [110] to detect the best-fitting model of molecular evolution for this dataset, which was determined to be a WAG + R5 model [111], according to the Bayesian Information Criterion [112]. A Maximum Likelihood (ML) phylogenetic inference analysis was subsequently run with IqTree [113]. The reliability of the generated tree was tested with 1000 ultrafast bootstrap replicates. Due to the lack of suitable outgroups, the tree was graphically represented as an unrooted tree.

### 3.4. Creation and Validation of Mytilectin-Specific Profile HMMs

The MSA of all mytilectins was modified to remove highly similar sequences (those displaying pairwise homology > 90%) to reduce the bias linked with the over-representation of mytilectin sequences from phylogenetically close taxa. The MSA was split in two parts, reflecting the position of the N-terminal β-trefoil and C-terminal pore-forming domains, respectively, as previously defined by other studies [88]. N-terminal and C-terminal extensions were removed, whenever present. The two profile HMMs were built with the HMMer hmmbuild module [114], and the hmmsearch module was used for testing their performance against two distinct datasets. Dataset (a) included all the mytilectin sequences previously described in the literature [20,21,26,88,115], and dataset (b) included the complete Uniprot sequence database [116]. Positive matches were detected using default e-value thresholds.

The “mytilectin N-terminal domain” and “mytilectin C-terminal domain” HMMs are available as Supplementary Materials S2 and S3, respectively.

## 4. Conclusions

Although previous studies have already highlighted the potential biotechnological use of mytilectins [26,30,115], so far, all available functional data concern a small number of sequences isolated from bivalve mollusks, whose biological role in the context of innate immunity still remains rather elusive. We reported here an overview of the distribution of this type of lectins in the animal kingdom, formally defining a widespread gene family. We noted a very ancient evolutionary origin and a complex distribution pattern characterized by a great number of gene loss events that occurred independently of each other in different phyla, also identifying the chimera-type architecture as the most ancestral one, from which proto-type mytilectins likely derived following the loss of the pore-forming domain. Although unusual, this patchy taxonomic distribution mirrors that previously described for other effectors of innate immunity in invertebrate organisms [117]. Understanding which evolutionary factors underlie the maintenance and loss of mytilectins in different taxa is a prerequisite for better understanding their biological role and improving their functional study, which will be made easier with the availability of profile HMMs specifically developed for their identification in large sequence databases.

## Data Availability

All mytilectin protein sequences are available in Supplementary Material S1.

## Supplementary Materials

The following supporting information can be downloaded at: https://www.mdpi.com/article/10.3390/md21120614/s1, File S1: amino acid sequences of all the mytilectins reported in this study (note that “G” and “T” indicate sequences identified from genomes and transcriptomes, respectively); File S2: profile HMM for the mytilectin N-terminal domain; File S3: profile HMM for the mytilectin C-terminal domain.
